# Expanding the known distribution of phascolartid gammaherpesvirus 1 in koalas to populations across Queensland and New South Wales

**DOI:** 10.1038/s41598-023-50496-4

**Published:** 2024-01-12

**Authors:** Belinda R. Wright, Andrea Casteriano, Yasmine S. S. Muir, Lyndal Hulse, Sarah J. Simpson, Alistair R. Legione, Paola K. Vaz, Joanne M. Devlin, Mark B. Krockenberger, Damien P. Higgins

**Affiliations:** 1 Sydney School of Veterinary Science, University of Sydney, Camperdown, NSW 2006 Australia; 2 School of Agriculture and Food Sciences, The University of Queensland, St Lucia, QLD 4072 Australia; 3 Melbourne Veterinary School, Faculty of Science, Asia Pacific Centre for Animal Health, University of Melbourne, Parkville, VIC 3010 Australia

**Keywords:** Biological techniques, Molecular biology

## Abstract

Koala populations across the east coast of Australia are under threat of extinction with little known about the presence or distribution of a potential pathogen, phascolartid gammaherpesvirus 1 (PhaHV-1) across these threatened populations. Co-infections with PhaHV-1 and *Chlamydia pecorum* may be common and there is currently a limited understanding of the impact of these co-infections on koala health. To address these knowledge gaps, archived clinical and field-collected koala samples were examined by quantitative polymerase chain reaction to determine the distribution of PhaHV-1 in previously untested populations across New South Wales and Queensland. We detected PhaHV-1 in all regions surveyed with differences in detection rate between clinical samples from rescued koalas (26%) and field-collected samples from free-living koalas (8%). This may reflect increased viral shedding in koalas that have been admitted into care. We have corroborated previous work indicating greater detection of PhaHV-1 with increasing age in koalas and an association between PhaHV-1 and *C. pecorum* detection. Our work highlights the need for continued surveillance of PhaHV-1 in koala populations to inform management interventions, and targeted research to understand the pathogenesis of PhaHV-1 and determine the impact of infection and co-infection with *C. pecorum.*

## Introduction

Koala populations across the eastern Australian states of New South Wales (NSW), Queensland (Qld) and the Australian Capital Territory (ACT) have been recently declared endangered^1^ with infertility, blindness and urinary tract disease due to *Chlamydia pecorum* infection being a major contributor to population declines^2,3^. Another important pathogen, koala retrovirus (KoRV), exists in both endogenous and exogenous forms, and with subtypes of varying prevalence detected across koala populations^4^. KoRV appears likely to cause neoplasia^5,6^ and is associated with greater severity or prevalence of chlamydia^7,8^, though causation is not proven. The impact of two recently identified gammaherpesviruses, phascolartid gammaherpesvirus 1 (PhaHV-1)^9^, and phascolartid gammaherpesvirus 2 (PhaHV-2)^10^, are less well understood, though significant associations have been identified between the PhaHVs and detection of *C. pecorum*^11,12^, and between PhaHV-1 infection and presence of KoRV^11^. Co-infections between these four koala pathogens may have compounding impacts on koala health, with further research required to determine these impacts and inform appropriate management actions.

Herpesviruses are double-stranded DNA viruses that are well known for establishing latency, with the site of latent infections being one of the distinguishing features of the three subfamilies, *Alpha*-, *Beta- and Gammaherpesvirinae*^13^. Gammaherpesviruses establish latent infections in B and T lymphocytes with active infections occurring in epithelial cells^14,15^. Detection of PhaHVs in koala liver and spleen^16,17^ suggests lymphocytes are also a likely site of latent PhaHV infection in koalas. In human and mouse studies, gammaherpesvirus infections have resulted in lymphoproliferative disorders in immunocompromised individuals^18^. Given the high prevalence of chlamydial infection in koala populations^19^, along with the potential immunosuppressive role of KoRV^20,21^, and its associations with neoplasia^5,22^, the impacts of co-infections with PhaHVs may play a significant role in koala health outcomes.

Research on the distribution and impacts of PhaHVs is currently limited to southern koala populations^11,12,17^. Of 80 wild-caught koalas from the Mount Lofty Ranges in South Australia, active shedding of PhaHVs were detected in 72.5%^17^. PhaHV-1 prevalence across Victorian populations ranges from 7.4% to 45.5%, with PhaHV-2 prevalence ranging from 0.9% to 54.6% (N ranges from 28 to 109 samples per population)^11^. Importantly, in Victorian populations, significant associations have been found between herpesvirus infection and infection with both *C. pecorum* and KoRV^11,12^. Comparisons between oropharyngeal swabs and spleen samples in euthanised koalas demonstrated that while 72.4% of koalas were systemically infected with PhaHVs, only 54% were actively shedding the virus, with PhaHV-1 actively shed in 48.9% of koalas and PhaHV-2 shed in 14.9%^17^. Active shedding of gammaherpesviruses can be stimulated by stress or co-infections^18,23^.

Although gammaherpesvirus infection has been identified as a negligible to moderate risk to koala populations, certainty on this evaluation is low, with the recent National Koala Disease Risk Analysis recommending increased research effort into the distribution of PhaHVs in northern koala populations^24^. To further identify the distribution and impacts of PhaHVs in koalas, we recently developed new diagnostic assays for PhaHV-1 infections^25^. Additional molecular assays for assessing PhaHV-2 are currently in development but are limited by lack of genomic sequence information for suitable target design. To date, only PhaHV-1 has been associated with increasing koala age and KoRV infections^11,17^ in Victorian and South Australian koalas. We here apply our new quantitative polymerase chain reaction (qPCR) assay to the detection of PhaHV-1 in the previously untested endangered populations of koalas across Qld and NSW. We provide valuable insights into the distribution of this important koala pathogen along with an assessment of factors that may be associated with PhaHV-1 detection, including chlamydial infection.

## Results

We surveyed 298 koala urogenital (UGT) swab samples from archived clinical and field-collected samples using qPCR^25^ and assessed relationships between detection of PhaHV-1, *C. pecorum*, age, sex and geographic region . We note that this sample type is likely to be detecting active shedding rather than latent infection which would require samples from a full necropsy. Samples comprised 247 clinical and 51 field-collected swabs from 137 males, 132 females and 29 of unknown sex. Age classes ranged from 1–7 and numbers of samples per population ranged from 5–26 (Table 1). The percentage of *C. pecorum* detected in samples across broad regions ranged from 8–59%.

**Table 1:** Samples surveyed from koala swabs across a range of regions with number of detections (+ve) for PhaHV-1 and *C. pecorum* (Cpec) using qPCR.

Region	No. of koalas	M	F	Unk	Age classes	PhaHV-1 + ve (%)	Cpec + ve (%)
St Bees Island Qld*	9	5	4	0	NA	1 (11)	0 (0)
Connors Range Qld*	16	4	12	0	NA	0 (0)	2 (13)
*Total*	*25*	*9*	*16*	*0*		*1 (4)*	*2 (8)*
South East Qld	23	7	16	0	I–V	7 (30)	9 (39)
Southern Qld†	16	12	3	1	II–V	4 (25)	9 (56)
North coast NSW	7	5	2	0	III–V	2 (29)	0 (0)
Northern Rivers NSW	20	12	7	1	I–VII	3 (15)	0 (0)
Northern NSW	13	4	8	1	II–V	5 (38)	5 (38)
*Total*	*79*	*40*	*36*	*3*		*21 (27)*	*23 (29)*
Northern Tablelands NSW	9	5	4	0	IV–V	2 (22)	6 (67)
Liverpool Plains	15	5	1	9	I–IV	7 (47)	0 (0)
Mid-north coast NSW	26	7	11	8	I–IV	6 (23)	8 (31)
*Total*	*50*	*17*	*16*	*17*		*15 (30)*	*14 (28)*
Central West NSW	24	17	7	0	I–V	4 (17)	7 (29)
Port Stephens	17	6	11	0	II–VI	6 (35)	7 (41)
Central Coast NSW/Hunter	5	4	1	0	II–V	4 (80)	3 (60)
Sydney region	26	14	12	0	I–V	5 (19)	8 (31)
Campbelltown*	10	6	4	0	I–IV	1 (10)	0 (0)
Southern Highlands†	15	8	7	0	I–III	2 (13)	2 (13)
Southern Tablelands NSW	20	8	10	2	I–VI	4 (20)	10 (50)
*Total*	*117*	*63*	*52*	*2*		*26 (22)*	*37 (32)*
South Australia	20	8	12	0	I–V	4 (20)	9 (45)
Victoria	7	0	0	7	NA	2 (29)	7 (100)
*Total*	27	8	12	7		6 (22)	16 (59)
*Grand total*	*298*	*137*	*132*	*29*	*I*–*VII*	*69 (23)*	*92 (31)*

Sub-totals are based on broad biogeographic regions. See Supplementary Table 1 for detailed data across all 298 samples.

*Samples were wild caught.

^†^Samples mixture of wild caught and rescue. All other samples were from archived clinical samples from rescued koalas. *M* male, *F* female, *Unk* unknown sex. PhaHV-1 results are from current study, Cpec (*C. pecorum*) are from previous clinical data.

**Figure 1:**
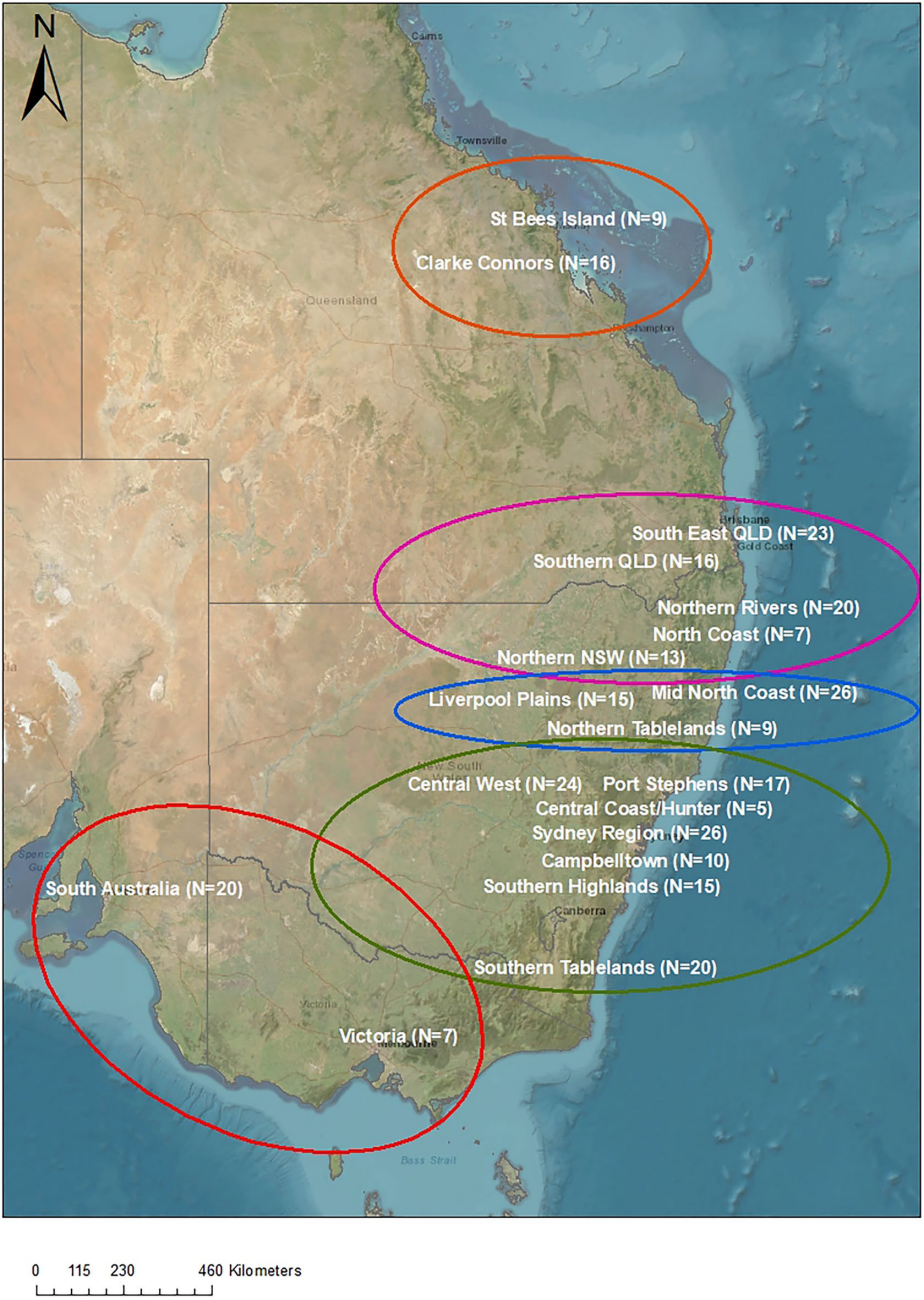
Sampling locations and numbers of koalas used in this study. Locations are approximate only as GPS co-ordinates were not available for most samples so location is based on submitting clinic. The five broad biogeographic areas are indicated by coloured circles: orange = Central QLD, pink = SE QLD/Northern NSW, blue = Mid-north NSW, green = Central/Southern NSW, red = Southern Australia.

**Figure 2:**
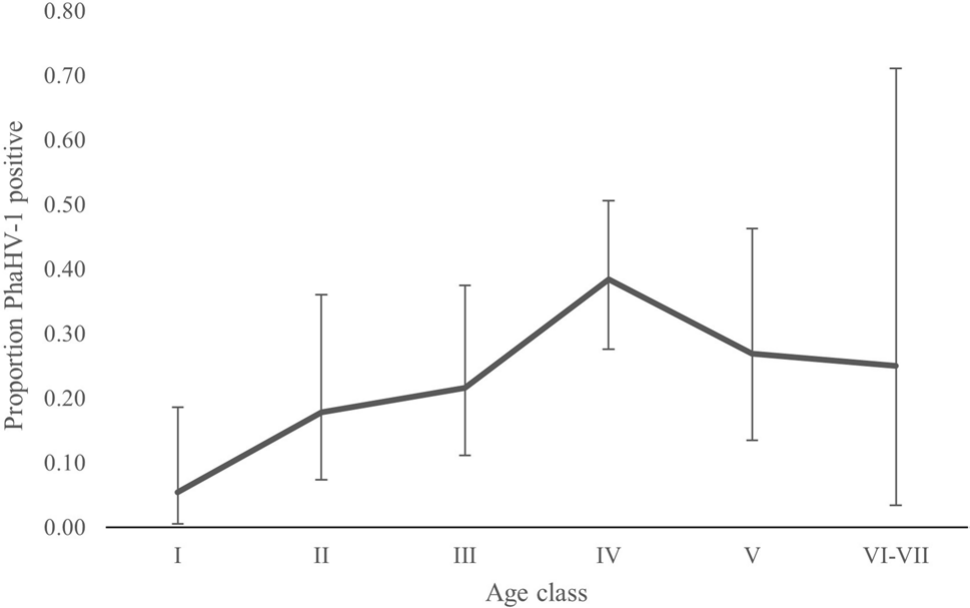
PhaHV-1 infection is more likely with increasing age class in koalas. Numbers of koalas in each age class: I (N = 37), II (N = 28), III (N = 37), IV (N = 65), V (N = 26), VI–VII (N = 4). Older age classes of VI and VII combined due to low numbers (2 in each). Error bars indicate 95% confidence intervals.

PhaHV-1 was detected in all populations sampled except the population of Clarke Connor’s Range in Central Qld (Table 1). From 298 samples surveyed using the PhaHV-1 qPCR assay, 69 (23%) were positive. As our sampling strategy was not appropriate for accurate assessment of PhaHV-1 prevalence across regions, we grouped samples into five broad biogeographic regions (see Methods) and found a similar prevalence among them with the exception of Central Qld (Fig. 1). Detection of PhaHV-1 was nearly four times more likely among *C. pecorum*-positive koalas than *C. pecorum*-negative (38/92, 41%; vs 31/206, 15%; *X*^2^ = 23.19, df = 1, p < 0.001; Odds ratio = 3.97, 95% CI = 2.26—6.98). Generalised linear model (GLM) results indicate PhaHV-1 is associated with increasing age and *C. pecorum* infection but not sex or bioregion (Tables 2 and 3a). PhaHV-1 detection was more likely in older animals than younger animals (Table 2, Fig. 2).

**Table 2:** Results of GLMs investigating factors predicting PhaHV-1 infection using *C. pecorum*, age and sex as predictors (N = 197).

Predictor	Estimate (95% CI)	Std. Error	P value
*(Intercept)*	*− 2.8670 (− 4.02 to 1.84)*	*0.5526*	< *0.0001*
*C. pecorum*	1.3578 (0.67 to 2.07)	0.3571	**0.0001**
Age	0.3622 (0.10 to 0.64)	0.1357	**0.0076**
Sex	*− *0.0865 (*− *0.80 to 0.61)	0.3589	0.8095

Coefficient estimate and standard error are presented. Significant p values are in bold.

Nb. Model averaging was not possible for this model as there was only one top model. Confidence intervals (CI) do not include 0 for significant predictors.

**Table 3:** **a.** Results of GLMs investigating factors predicting PhaHV-1 infection using *C. pecorum* and broad geographic bioregion as predictors (N = 298). Top two AIC_C_ used in final models: 311.6, 313.43. Coefficient estimate and adjusted standard error are presented. Significant p values are in bold. **b.** Modelling results for 73 *C. pecorum* positive koalas with data available on clinical signs, using PhaHV-1 infection and sex as predictor variables. Age was included for a subset of 59 koalas but did not appear in final models so is not presented. Top two AIC_C_ used in final models: 94.32, 95.11.

Predictor	Estimate (95% CI)	Adjusted SE	RI	N. models	P value
**a.*** (Intercept)*	*0.2075 (0.09–0.33)*	*0.0604*			*0.0006*
*C. pecorum*	0.2629 (0.16*–*0.36)	0.0514	1	2	** < 0.0001**
Bioregion *					
Central Qld	− 0.0135 (− 0.27 to 0.18)	0.0654	0.29	1	0.8369
SE Qld	0.0352 (− 0.06 to 0.30)	0.0742	0.29	1	0.6351
Mid NSW	0.0458 (− 0.03 to 0.35)	0.0894	0.29	1	0.6085
Southern NSW	0.0208 (− 0.10 to 0.24)	0.0573	0.29	1	0.7163
**b.** *(Intercept)*	*0.0351 (*− *0.48 to 0.55)*	*0.2597*			*0.8925*
PhaHV-1	0.5356 (− 0.16 to 1.95)	0.6040	0.6	1	0.3752
Sex	1.5619 (0.54*–*2.59)	0.5209	1	2	**0.0027**

*Southern Australia used as the reference level for broad bioregion. Each region was assessed as the reference level for comparison and no significant effects of Bioregion were found (data not shown). Relative importance (RI) and number of top models (N. models) is for Bioregion as a single factor. Confidence intervals (CI) do not include 0 for significant predictors.

Detection of PhaHV-1 was 4.2 times more likely in the rescued cohort of koalas relative to the free-living cohort (65/247, 26% vs 4/51, 8%;* X*^2^ = 7.102, df = 1, p-value = 0.008; Odds ratio = 4.20, 95% CI = 1.45—12.11). Age class ranges were similar across the rescued and free-living cohorts (Table 1) suggesting that age differences between the two cohorts is not driving the difference in PhaHV-1 detection (see Methods for cohort distinctions). Data on age were not available for all samples and were missing for a greater proportion of free-living samples (Table 1), so we cannot make an accurate comparison of age differences between the two cohorts. For 73 *C. pecorum* known-positive koalas, clinical signs (consistent with chlamydial disease) were not associated with detection of PhaHV-1 or age, but were associated with sex (Table 3b). Of these 73 koalas, females were more likely than males to present clinical signs (Males 12/37, 32%; Females 25/36, 69%; *X*^2^ = 8.57, df = 1, p = 0.003; Odds ratio = 4.73, 95% CI = 1.76, 12.72). Of 230 koalas with data recorded on clinical signs, 81 were observed to display clinical signs. Sex differences were evident in the site of clinical signs with 24 males and 14 females showing ocular signs, and 13 males and 30 females showing urogenital signs.

## Discussion

We have identified PhaHV-1 in all regions sampled in this study extending the known range of this potential pathogen into the endangered koala populations of NSW and Qld. Although the opportunistic nature of sampling meant that sample numbers were small and biased towards rescued koalas in many populations, low detection of PhaHV-1 in some populations warrants further investigation. We have corroborated previous work that found an association between PhaHV-1 detection and increasing age in koalas from Victoria and South Australia^11,17^. Likewise, our study confirms previous work identifying an association between PhaHV-1 and chlamydia in southern populations^11,12^. Detection of PhaHV-1 was not associated with presentation of clinical signs in koalas also testing positive for *C. pecorum* but this analysis was limited by incomplete metadata in clinical and field-collected records.

Due to the opportunistic and the largely rescue-sourced nature of sampling in our study, we are unable to determine precise prevalence of PhaHV-1 across free-living koala populations. The similarity in percentage of PhaHV-1 detection (22 – 30%) found among rescued koalas across the broad biogeographic regions does not necessarily indicate homogeneity within those regions and further targeted sampling effort is required to determine if differences exist in prevalence between free-living vs rescued koalas. For example, two populations from the Mackay region of Central Qld (St Bees Island and Connors range to the west of Mackay) had low PhaHV-1 detection frequency, as did the Campbelltown population in the Sydney region, which was assessed separately to other Sydney region populations as it is currently considered to be *Chlamydia*-free^26^ and potentially isolated. All three populations were sampled during field research rather than by clinical sampling following rescue. Previous work has also found regional differences in PhaHV-1 prevalence, with lower (8.4%) than average (17.4%) prevalence in the closed French Island population^11^ which also has a very low prevalence of chlamydia^27^.

The higher proportion of PhaHV-1 detection in koala samples from rescued koalas in comparison to free-living koalas may also indicate higher shedding of the virus from sick or injured koalas, and/or increased stress due to being held in captivity for treatment. The qPCR assay and sample types used in the current study are not suited to detection of latent infection, and previous studies have shown only 48.9% of koalas infected with PhaHV-1 to be actively shedding the virus^17^. In human studies *Chlamydia trachomatis* infection can induce replication of latent Human herpesvirus-6 infections^28^. More research is needed to understand the mechanistic relationship between *C. pecorum* and PhaHV infections. Likewise, while the current study supports previous work identifying an association between PhaHV-1 and *C. pecorum* infection^11,12^, this association may indicate activation and shedding of PhaHV-1 as a result of chlamydial infection, detection of latent virus in cells exuded as a result of chlamydial inflammation, or greater shedding of both due to an unidentified underlying mechanism or co-infection. A targeted study of the impact to koalas health of coinfections by *C. pecorum*, PhaHV-1 and KoRV is needed, but was beyond the scope of the current study as data on clinical signs and coinfections were not available for all samples limiting the power of analyses.

Our findings support previous work demonstrating an increasing prevalence of PhaHV-1 and increasing age in koalas^11,17^. The low prevalence in younger age classes may indicate that acquiring PhaHV-1 infection occurs as koalas mature and engage in sexual or aggressive contact^11,17^. We did not find an association between sex and PhaHV-1 detection, and past research has been equivocal on this^11,12,17^. Stalder et al.^12^ found PhaHV-1 more likely to be detected in male koalas, while Vaz et al.^11^ and Kasimov et al.^17^ found no association with sex. Vaz et al.^11^ found females without young to be 1.7 times more likely to be infected with PhaHV-1 than females with young. Further research should use a targeted sampling approach across age classes, balanced sex ratios and over time and breeding seasons to fully understand transmission dynamics of PhaHVs.

PhaHV-1 infection has been associated with clinical signs^11^, but we did not find an increased likelihood of clinical signs in koalas with both *C. pecorum* and PhaHV-1 detected. The available metadata was not consistent across samples and had limited detail, so we were not able to assess any association between coinfections and severity of clinical signs. Clinical signs were more commonly recorded in female koalas though this was likely influenced by the higher proportion of urogenital clinical signs recorded in our dataset. Male koalas are more likely to present ocular signs and female koalas are more likely to present urogenital signs^29,30^. As our samples and metadata were obtained from a wide variety of sources, we cannot confirm that procedures were the same across facilities but have controlled for sample quality by testing for the koala β-actin gene. The associations between PhaHV-1, chlamydia and KoRV^11,12^ necessitates careful sampling and standardised recording of metadata to enable future studies to resolve uncertainties relating to factors influencing co-infections, causal associations and clinical outcomes for koalas.

Our study has confirmed the widespread presence of PhaHV-1 in koala populations across NSW and Qld, and across biogeographic barriers, contributing to our understanding of exposure risk in disease risk analyses and laying the basis for future work investigating prevalence of PhaHV-1 infection in these regions. The wide distribution, potential differences in prevalence of PhaHV-1 among populations and free-living versus rescued cohorts, and the limited understanding of the impact of PhaHV-1 and other co-infections on koala health and clinical presentations highlights the need for further research and precautionary routine surveillance of PhaHV-1 prior to management interventions in wild, rehabilitation and captive koala populations.

## Methods

### Samples

We examined a total of 298 koala samples including 247 clinical samples from the Koala Health Hub sample archive (University of Sydney), consisting of previously extracted DNA from UGT swabs submitted for clinical chlamydial diagnostics. These samples were obtained opportunistically and collected as part of routine clinical examination from rescued koalas in care at veterinary hospitals and wildlife care clinics and are subsequently referred to as the “rescued cohort”. The majority of clinical samples were collected between 2017 and 2022, with seven samples from Victoria collected between 2010 and 2015. The remaining archived samples (*n* = 51) were collected during previous field surveys (prior to the current study) in the Southern Highlands and Campbelltown region of NSW (collected between 2021 and 2022) and three wild populations across Qld located at St Bees Island and Clarke Connors range in Central Qld and Oakey in Southern Qld (collected between 2016 and 2017) and are subsequently referred to as the “free-living cohort” (Fig. 1). All methods are reported in accordance with ARRIVE guidelines ^31^ and were carried out in accordance with relevant guidelines and regulations. All experimental protocols were approved by the relevant institutions (University of Sydney Ethics approval 2019–1547, University of Queensland Ethics approval CMLR/304/13/QLD GOVT and CMLR/091/12/ARC/RIO TINTO, Central Queensland University Animal Ethics Approval A72/04–282, and Scientific Purposes permits WISP16162915 and WISP15517315). We selected urogenital swabs because evidence is equivocal as to whether urogenital or oropharyngeal swabs are more sensitive for PhaHV-1 detection^11,17,25^, and one of our aims was to investigate correlations with chlamydial infection as determined from UGT swabs. Archived samples were selected to represent a wide geographic area of the koalas’ range, especially from previously untested regions in Qld and NSW, as well as 27 samples from southern populations for comparison (Table 1). As sampling was opportunistic, and largely biased to clinical samples from koalas admitted to care, we note that our assessment of PhaHV-1 prevalence is not reflective of a random sample of free-ranging koalas.

Quantitative PCR assays were applied as previously described^25^ using a koala β-actin gene qPCR^32^ as a sample quality control to ensure sample integrity and sufficient DNA present in the sample for amplification. All clinical samples had been tested for *C. pecorum* infection status using previous diagnostic assays^32–35^. Data on age, sex and clinical signs were available for the majority of samples (Table 1). Koala age was grouped into age classes based on reported tooth wear as follows: I (1–2 yrs), II (2–3 yrs), III (4 yrs), IV (5–6 yrs), V (10–12 yrs), VI (12 + yrs), VII (15 + yrs)^36,37^. Data on clinical signs were very general only and consisted of indications of either clinical signs consistent with chlamydial disease being urogenital signs, ocular signs or both, or no clinical signs present. Only broad age categorisations were available for wild caught koalas from the three Qld populations, but the majority (32/36, 89%) were classified as adults.

### Statistical analyses

We used generalised linear models (GLMs) to investigate the relationships between detection of PhaHV1 and infection with *C. pecorum* (Cpec), age, sex and region. PhaHV-1 detection was the binary response variable using the binomial family in R (v 4.2.0)^38^. As our sampling strategy did not allow population-level assessment of PhaHV-1 prevalence, koala (N = 298) sampling locations were grouped into five broad regions based on previously identified biogeographic barriers on the east coast and historical translocations impacting phylogenetics of southern populations^39,40^. Data on age and sex were available for 197 koalas so these variables were included as potential predictors of PhaHV-1 detection for these samples. As we were interested in whether co-infection would increase the likelihood of presenting clinical signs, we studied a further subset of koalas that had tested positive for *C. pecorum* and had data on clinical signs consistent with chlamydiosis (N = 73). Clinical signs consisted of observational recording of urogenital and/or ocular signs of potential disease and as the level of detail varied across records this was reduced to a binary term. We conducted GLMs using clinical data (binary; yes, no) as the response variable and PhaHV-1 detection (HV1) and sex as potential explanatory variables. Age was included as a factor for a subset of 59 *C. pecorum* positive koalas. GLMs were conducted in R^38^, using the ‘LMe4’ package^41^. We employed an information theoretic approach to identify the best models using model averaging following Grueber et al.^42^. This approach first standardises models using the ‘arm’ package^43,44^, then uses the top two AIC_C_ (small sample-size corrected Akaike information criterion) of models using the ‘MuMIn’ package^45^ to generate final models. The relative importance (RI) of each explanatory variable was calculated by summing the Akaike weight of each of the final models that the predictor appeared in, with RI of 1 being indicative of a strong predictor. We also conducted univariate analyses to obtain p values and 95% confidence intervals via odds ratios and *X*^2^ using 2 × 2 tables for significant predictors identified using GLM.

### Supplementary Information

Supplementary Information.

## Data Availability

Complete dataset is available in Supplementary Table 1.
